# Induced neural stem/progenitor cell‐derived extracellular vesicles promote recovery post‐stroke

**DOI:** 10.1002/ctm2.936

**Published:** 2022-06-22

**Authors:** Ge Gao, Congcong Li, Jie Zhu, Shiyang Sheng, Zhanping Liang, Shengyang Fu, Xiangyu Li, Yiyan Sun, Yi Wang, Xuanran Feng, Xiaohuan Xia, Jialin C. Zheng

**Affiliations:** ^1^ Center for Translational Neurodegeneration and Regenerative Therapy Tongji Hospital affiliated to Tongji University School of Medicine Shanghai China; ^2^ Center for Translational Neurodegeneration and Regenerative Therapy Shanghai Tenth People's Hospital affiliated to Tongji University School of Medicine Shanghai China; ^3^ Translational Research Center Shanghai Yangzhi Rehabilitation Hospital affiliated to Tongji University School of Medicine Shanghai China; ^4^ Shanghai Frontiers Science Center of Nanocatalytic Medicine Tongji University Shanghai China; ^5^ Translational Research Institute of Brain and Brain‐Like Intelligence Shanghai Fourth People's Hospital Affiliated to Tongji University School of Medicine Shanghai China; ^6^ Collaborative Innovation Center for Brain Science Tongji University Shanghai China

To the editor,

Ischemic stroke (IS) is one of the leading causes of mortality and disability worldwide.^1^ Among various therapeutic strategies for IS, the application/administration of stem cell‐derived extracellular vesicles (EVs), heterogeneous populations of small bilipid layer‐enclosed vesicles, has emerged as a promising one.^2^, ^3^ The administration of EVs derived from stem cells obtain similar neuroprotective and neuroregenerative effects to stem cell transplantation post IS.^3^ More importantly, compared to cell‐therapy, EV‐based therapy is with low immunogenicity, more flexible administration options, no vascular obstructive effect, reduced risk of secondary microvascular thrombosis, and ability to surface and content modification.^3^ Although mesenchymal stromal cells (MSCs) are the most commonly used stem cells for EVs generation, studies have demonstrated that neural stem/progenitor cell (NPC)‐derived EVs (NPC‐EVs) have better effects in improving post‐stroke recovery than MSC‐derived EVs.^4^ However, the application of NPCs as EVs producers is struggled with many concerns including religious/ethical questions and problematic logistics of acquiring fetal tissues, which can be overcame by reprogramming somatic cells into induced NPCs (iNPCs).^5^ Our previous studies found that iNPC‐EVs are with proliferation and cell survival promotion potential no less than NPC‐EVs in vitro, indicating iNPC‐EVs as eligible substitutes of NPC‐EVs.^6^, ^7^

Herein, we compared the therapeutic effects of NPC‐EVs and iNPC‐EVs on a model of IS using transient middle cerebral artery occlusion (MCAO) mice. MCAO was validated by triphenyltetrazolium chloride staining (Figure S1). NPCs were isolated from brain tissues of embryonic day 13.5 mice. iNPCs were generated by direct reprogramming of astrocyte via transgene approach.^5^ EVs were isolated from NPC and iNPC culture medium via ultracentrifugation, and characterized by western blotting, NTA analyses, and transmission electron microscopy (Figure S2). Indocyanine green‐labeled EVs were injected intravenously and could be observed in whole mouse body and mouse brains including brain 5 min after administration (Figure S3). Moreover, we analyzed the brain distribution of EVs after intravenously injecting Dil‐labeled NPC‐ and iNPC‐EVs. Dil signals were co‐localized with the immunoreactivities of GFAP (Figure S4A), Iba1 (Figure S4B), and Tuj1 (Figure S4C), implying the uptake of NPC‐ and iNPC‐EVs by glial and neuronal cells in mouse brains. We then assessed the sensorimotor functions of MCAO mice (Figure 1A). We examined the forelimb grip strength of the mice using hanging wire test, where both NPC‐ and iNPC‐EV‐injected MCAO mice significantly outperformed control MCAO animals (Figure 1B). The rotarod test also showed alleviation in both NPC‐ and iNPC‐EVs injection groups, which outperformed the control MCAO groups (Figure 1C). Similarly, the adhesive removal test demonstrated significant reduction of the tape‐remove times in both NPC‐ and iNPC‐EVs injection groups versus MCAO controls (Figure 1E). Interestingly, only iNPC‐EV‐injected mice showed significantly shorter tape‐contact times than MCAO controls (Figure 1D). Furthermore, we observed long‐term improvements in cognitive function of iNPC‐EV‐injected MCAO mice, but not NPC‐EV‐injected ones, in the morris water maze test versus MCAO controls, including a significantly reduction in the latency to find the platform (Figure 1F,G), increased platform crossing numbers (Figure 1F,H), and more distance traveled/time spent in the target quadrant when the platform was removed (Figure 1I,J). No difference in swimming speed among different groups was observed, indicating similar gross motor skills across groups (Figure 1K). Overall, our results demonstrated that iNPC‐EVs displayed comparable therapeutic effects as NPC‐EVs in promoting sensorimotor function recovery and higher capacity in cognitive function improvement than NPC‐EVs post MCAO.

**FIGURE 1:**
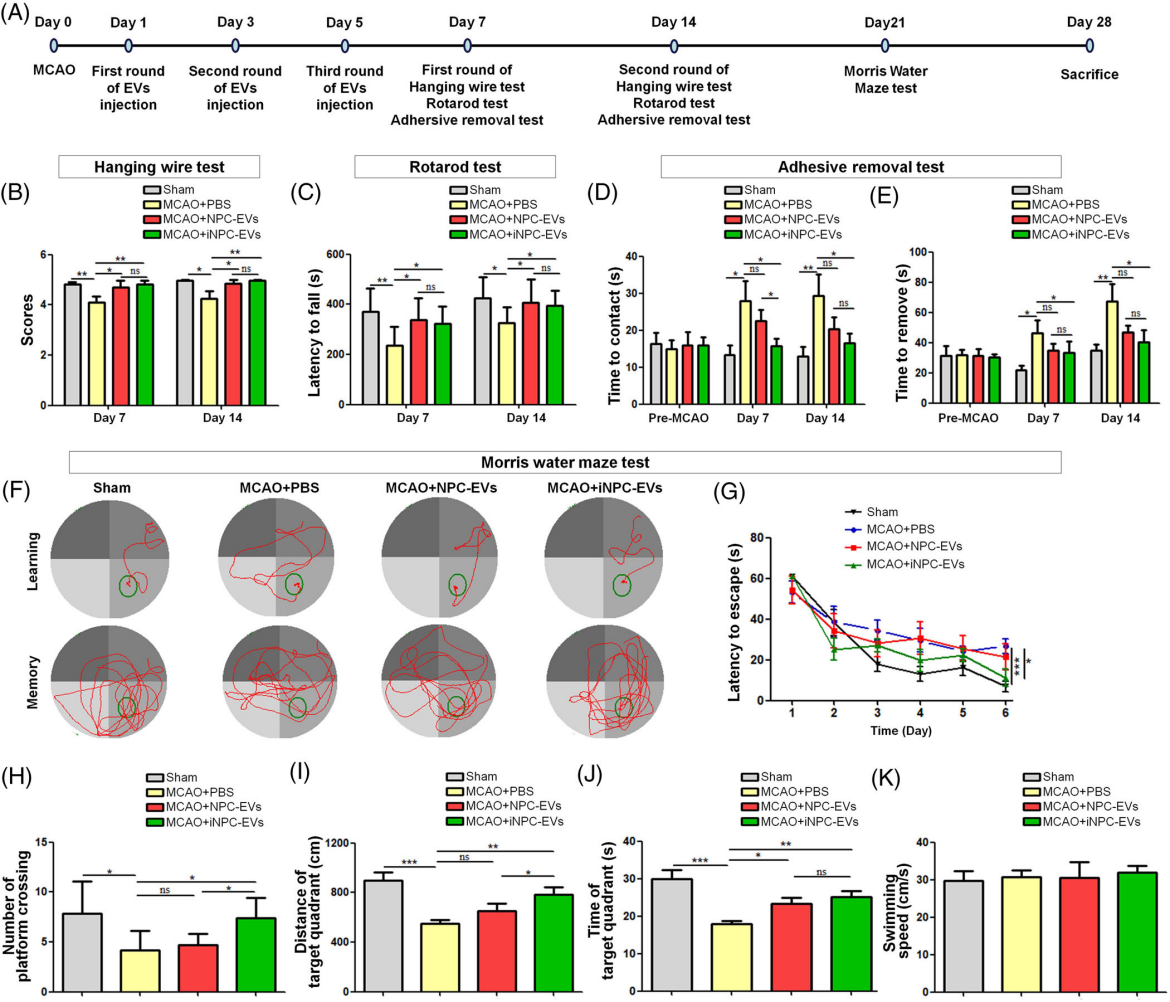
Post‐stroke administration of induced neural stem/progenitor cell (iNPC)‐extracellular vesicles (EVs) improves neurological functions after middle cerebral artery occlusion (MCAO). Mice received intravenous injection of 300 μl EVs (0.5 μg/μl) or PBS 24 h after 30‐min MCAO and at 2 days intervals on days 1–5 after stroke. (A) Experimental design. (A–E) Sensorimotor functions were assessed up to 14 d after MCAO or sham operation via hanging wire test (B), rotarod test (C), and adhesive removal test (D and E). (F–K) Long‐term cognitive functions were evaluated in the Morris water maze. (F) Representative images of the swim paths of mice in each group. The escape times (G, learning), the time spent (H)/distance traveled (I)/platform crossing numbers (J) in the target quadrant (memory), and the swim speed (K) were recorded. *n* = 9–13/group. Error bars denote s.d.. *p < .05, **p < .01, ***p < .001, and ****p < .0001. ns denotes non‐significance. The statistical difference among groups was assessed with the parametric one‐way the analysis of variance (ANOVA) with post hoc Bonferroni test.

Next, we examined the effects of both NPC‐ and iNPC‐EVs on the histopathological changes post MCAO. Both EVs increased the proportions of Sox2^+^ and Ki67^+^ cells in the hippocampus (Figure 2A), peri‐infarct cortex (Figure 2B), and sub‐ventricular zone (SVZ) (Figure 2C) of EV‐injected MCAO mice versus that of control MCAO mice, indicating a positive role of EVs on the expansion of NPC pool and the proliferation of NPCs post MCAO. Both NPC‐ and iNPC‐EVs restored the loss of neurons in the hippocampus (Figure 2D), cortex (Figure 2E), and SVZ (Figure 2F) of MCAO mouse brains, ascertained by the significant elevation of proportions of NeuN^+^ and DCX^+^ cells. The significantly higher proportions of NeuN^+^ cells might also indicate the neuroprotection effects of EVs in the initial phase of post‐stroke. Therefore, both EVs enhance neurogenesis post MCAO, and iNPC‐EVs may be with higher efficacy in promoting NPC proliferation (Figure 2B) and neuroregeneration in the MCAO brain (Figure 2D–F). Besides, western blotting results indicated significant down‐regulation and up‐regulation of the expression levels of pro‐inflammatory factor Cox2 and anti‐inflammatory protein MRC1, respectively, in the hippocampal (Figure 3A) and peri‐infarct cortical (Figure 3B) tissue lysates of EV‐injected MCAO mice, compared with that of control mice. EV‐injected mice also showed a significant decline in the proportions of Iba1^+^ cells in the hippocampus (Figure 3C) and peri‐infarct cortex (Figure 3D) versus control mice, suggesting comparable anti‐inflammatory effects of both NPC‐ and iNPC‐EVs post MCAO. Furthermore, the administration of EVs abrogated the MCAO‐induced down‐regulation of anti‐apoptotic *Bcl2* expression in the hippocampus (Figure 3E) and peri‐infarct cortex (Figure 3F). The injection of iNPC‐EVs but not NPC‐EVs inhibited pro‐apoptotic *Casp3* and *Casp8* expression in tested brain regions (Figure 3E,F). The TUNEL assay also showed anti‐apoptotic effects of EVs in the MCAO mouse hippocampus, suggesting that both NPC‐ and iNPC‐EVs promote cell survival in the MCAO mouse brain, and iNPC‐EVs exhibited higher anti‐apoptotic potential than NPC‐EVs (Figure 3G,H). We also examined the levels of pro‐inflammatory cytokines TNF‐α and IL‐6 in mouse sera 3 days after intravenous injection of EVs via ELISA assay. No significant difference has been observed among PBS‐, NPC‐EV‐, and iNPC‐EV‐injected mice and control ones, suggesting minor effects of EVs on peripheral immune system (Figure S5). It is worth‐noting that the long‐term therapeutic outcomes of NPC‐ and iNPC‐EVs post IS may be mediated, partially at least, by the shot‐term therapeutic effects of EVs, which needs to be determined in the future study. Thus, both behavioral test and histological/molecular studies suggested outstanding therapeutic potential of both NPC‐ and iNPC‐EVs in treating IS without significant adverse effect. Since promising results have been reported for the clinical safety of clinical‐grade EVs,^8^ the production and clinical safety of clinical‐grade iNPC‐EVs are required to be investigated in our future study.

**FIGURE 2:**
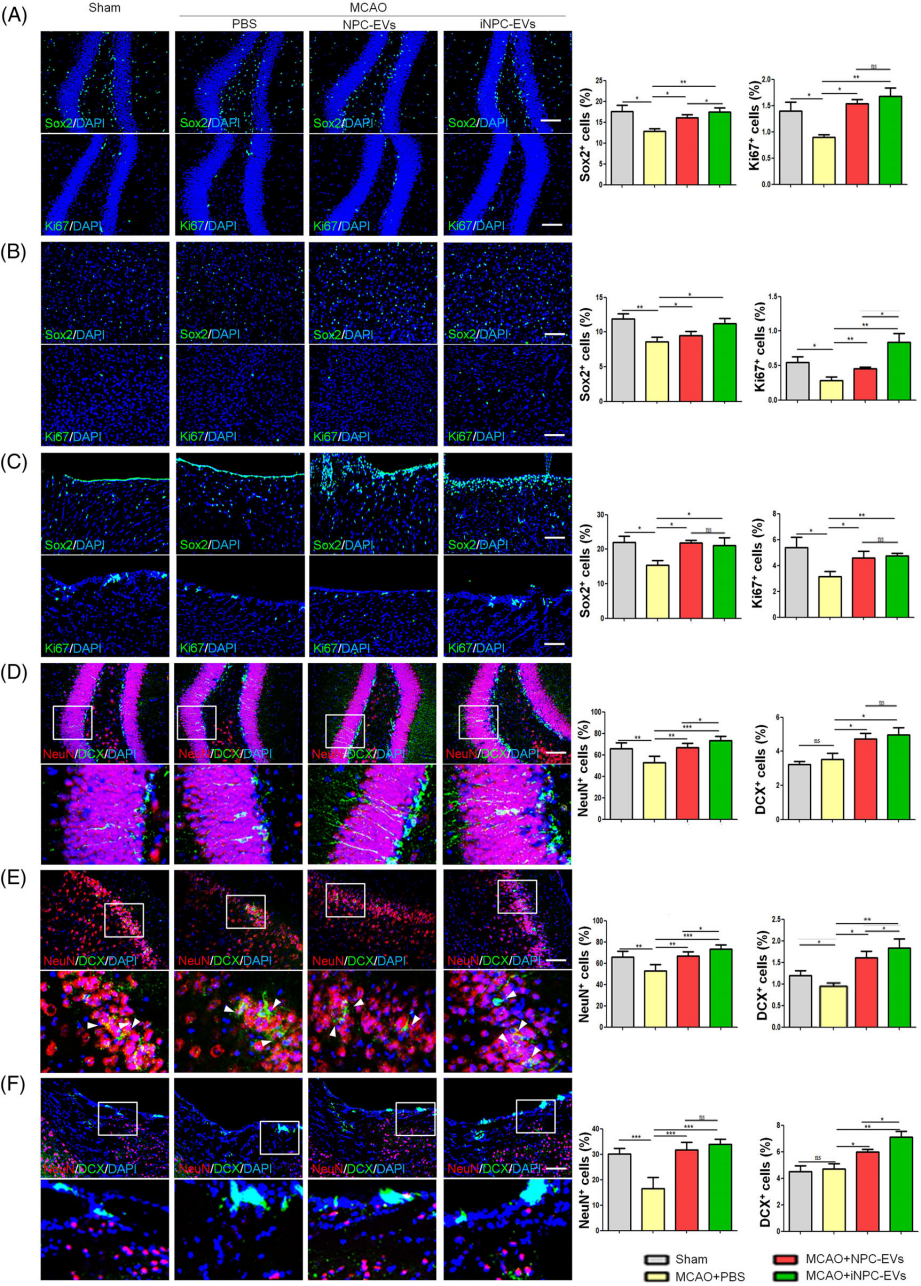
Post‐stroke administration of induced neural stem/progenitor cell (iNPC)‐extracellular vesicles (EVs) improves neurogenesis, inhibits neuroinflammation and represses apoptosis. Focal cerebral ischemic brains treated with or without EVs, and their sham controls were collected on day 28 after middle cerebral artery occlusion (MCAO). (A–C) Representative confocal microscopy images of Sox2 (green) and Ki67 (green) in the hippocampus (A), peri‐infarct cortex (B) and sub‐ventricular zone (SVZ) (C). Proportions of cells with Sox2 and Ki67 immunoreactivities in each group were given on the right panel (*n* = 4). (D–F) Representative confocal microscopy images of DCX (green) and NeuN (red) in the hippocampus (D), peri‐infarct cortex (E), and SVZ (F). Proportions of cells with DCX and NeuN immunoreactivities in each group were given on the right panel (*n* = 4). Scale bar: 100 μm. Error bars denote s.d.. *p < .05, **p < .01, ***p < .001 and ****p < .0001. ns denotes non‐significance. The statistical difference among groups was assessed with the parametric one‐way ANOVA with post hoc Bonferroni test

**FIGURE 3:**
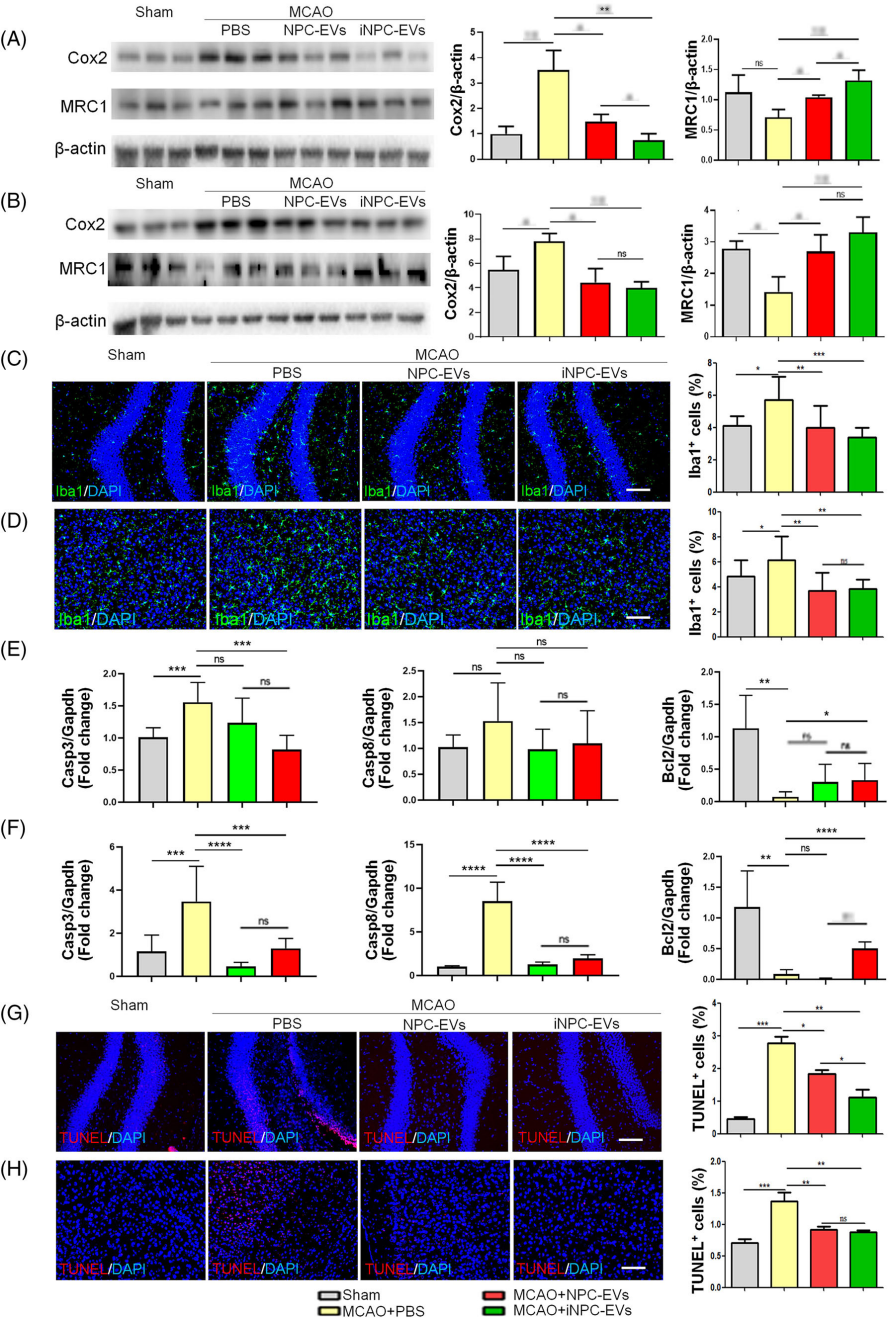
Post‐stroke administration of induced neural stem/progenitor cell (iNPC)‐extracellular vesicles (EVs) inhibits neuroinflammation. Focal cerebral ischemic brains treated with or without EVs, and their sham controls were collected on day 28 after middle cerebral artery occlusion (MCAO). (A and B) Representative blot (left) and quantification (right) of Cox2 and MRC1 protein expression levels in the hippocampus (A) and the peri‐infarct cortex (B) (*n* = 3). (C and D) Representative confocal microscopy images of Iba1 immunostaining (green) in the hippocampus (C) and the peri‐infarct cortex (D). Proportions of cells with Iba1 immunoreactivities were given on the right panel (*n* = 4). (E and F) Transcript levels of *Casp3*, *Casp8* and *Bcl2* in the hippocampus (**E**) and the peri‐infarct cortex (F) were determined by quantitative reverse transcription polymerase chain reaction (RT‐qPCR) analyses (*n* = 4). (G and H) Representative confocal microscopy images of TUNEL (red) staining in the hippocampus (G) and the peri‐infarct cortex (H). Proportions of TUNEL positive cells were given on the right panel (*n* = 4). RT‐qPCR data were normalized to *Gapdh* and presented as fold changes compared with sham control groups. Western blotting data were normalized to β‐actin. Scale bar: 100 μm. Error bars denote s.d.. *p < .05, **p < .01, ***p < .001, and ****p < .0001. ns denotes non‐significance. The statistical difference among groups was assessed with the parametric one‐way ANOVA with post hoc Bonferroni test

We then carried out preliminary studies to identify the potential mechanisms underlying the iNPC‐EV‐mediated recovery post MCAO. Since our proteomic analysis has implied enrichment of growth factor domains in iNPC‐EVs,^6^ we examined the protein levels of multiple growth factors that are commonly expressed in the brain cells in EVs. Western blotting results detected EGF, FGF2, and IGF2 in both EVs, and iNPC‐EVs contains higher levels of growth factors than NPC‐EVs (Figure S6). Both EVs activated MEK‐ERK pathway, but not PI3K‐AKT pathway, in both hippocampal and cortical tissues, ascertained by the up‐regulation of MEK and ERK phosphorylation (Figure S7). Moreover, iNPC‐EVs are with stronger promoting effects on the activities of MEK‐ERK pathway than NPC‐EVs (Figure S7). EV‐enhanced phosphorylation of ERK was further confirmed by immunohistochemistry, which displayed a significant increase of proportions of p‐ERK^+^ cells in both NPC‐ and iNPC‐EV treatment groups versus PBS controls (Figure S8). Given the important roles of MEK‐ERK signaling in the neurogenesis, inflammation, and apoptosis,^9^, ^10^ our preliminary results implied that EVs achieve their therapeutic effects via modulating MEK‐ERK signaling activities, and explained the potential mechanisms for the greater therapeutic effects of iNPC‐EVs.

In summary, iNPC‐EVs are with therapeutic effects no less than NPC‐EVs, suggesting iNPC‐EVs as an outstanding succedaneum of NPC‐EVs in treating IS. More importantly, since multiple histopathological features (e.g., neuroinflammation and neuronal damage/degeneration) are shared in neurological disorders, iNPC‐EVs may also achieve therapeutic effects on treating acute brain injury like traumatic brain injury and chronic neurodegenerative diseases, which is under investigation.

## CONFLICT OF INTEREST

The authors declare no competing commercial or financial interests.

## Supporting information

Supporting Information
