# Supplementary material for: Induced neural stem/progenitor cell‐derived extracellular vesicles promote recovery post‐stroke
Source: Clin Transl Med. 2022 Jun 22;12(6):e936. doi: 10.1002/ctm2.936 (PMC9217134; doi:10.1002/ctm2.936)
Supplement: Supplementary file 1 — Supporting Information [file CTM2-12-e936-s001.docx]

**Supplementary Information**

**Induced neural stem/progenitor cell-derived extracellular vesicles promote recovery post stroke**

**Ge Gao, Congcong Li, Jie Zhu, Shiyang Sheng, Yi Wang, Xuanran Feng, Shengyang Fu, Xiaonan Xu, Xiaohuan Xia, Jialin C. Zheng**

**Supplementary Outline**

Supplementary Figure 1

Supplementary Figure 2

Supplementary Figure 3

Supplementary Figure 4

Supplementary Figure 5

Supplementary Figure 6

Supplementary Figure 7

Supplementary Figure 8

Supplementary Table 1

Materials and methods

References

**
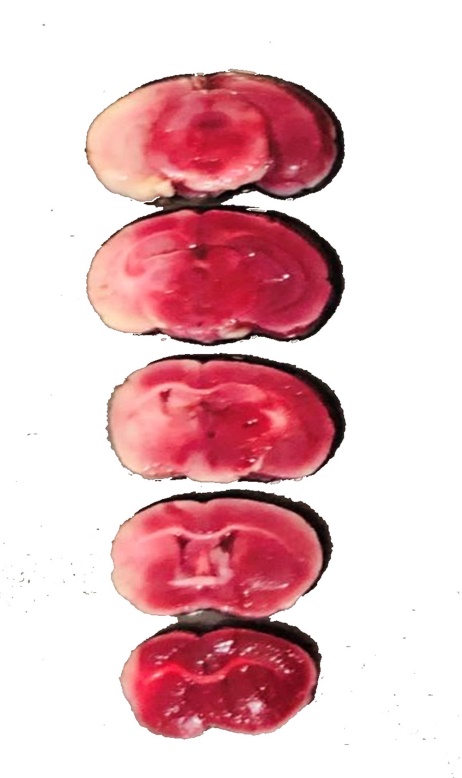
**

**Supplementary Figure 1. Validation of IS**.

TTC staining was performed using serial coronal slices of MCAO mouse brain, which showed red healthy zones and pale infarcted regions.


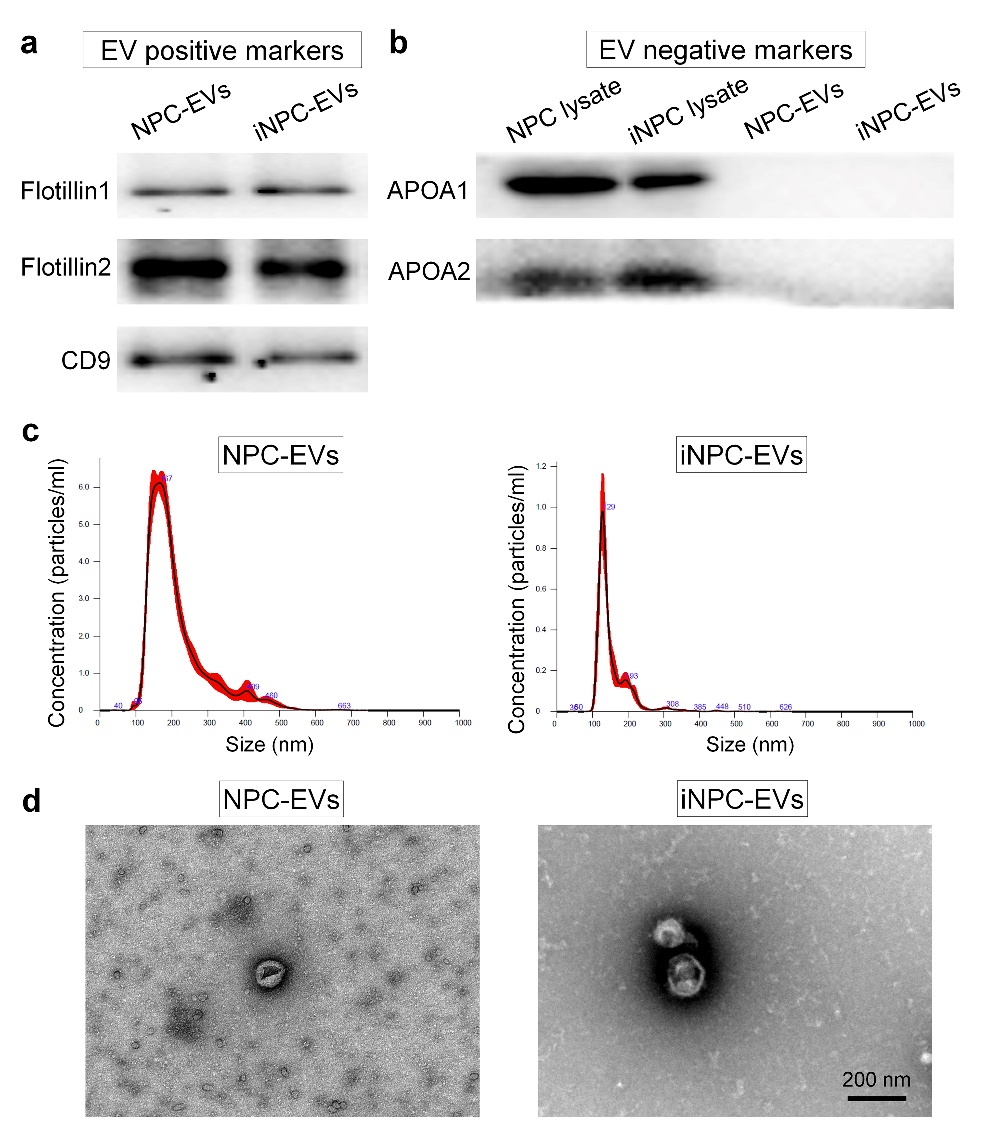


**Supplementary Figure 2. Characterization of EVs**.

(**a**) The expression of positive EV markers Flotillin1, Flotillin2, and CD9 in EVs were determined by western blotting. (**b**) The expression of negative EV markers APOA1 and APOA2 in EVs and cell lysates were determined by western blotting. (**c**) Particle-size distribution of EVs was determined by NTA. (**d**) Transmission electron microscopy characterization of the morphology of NPC-EVs and iNPC-EVs. Scale bar, 200 nm (**d**).


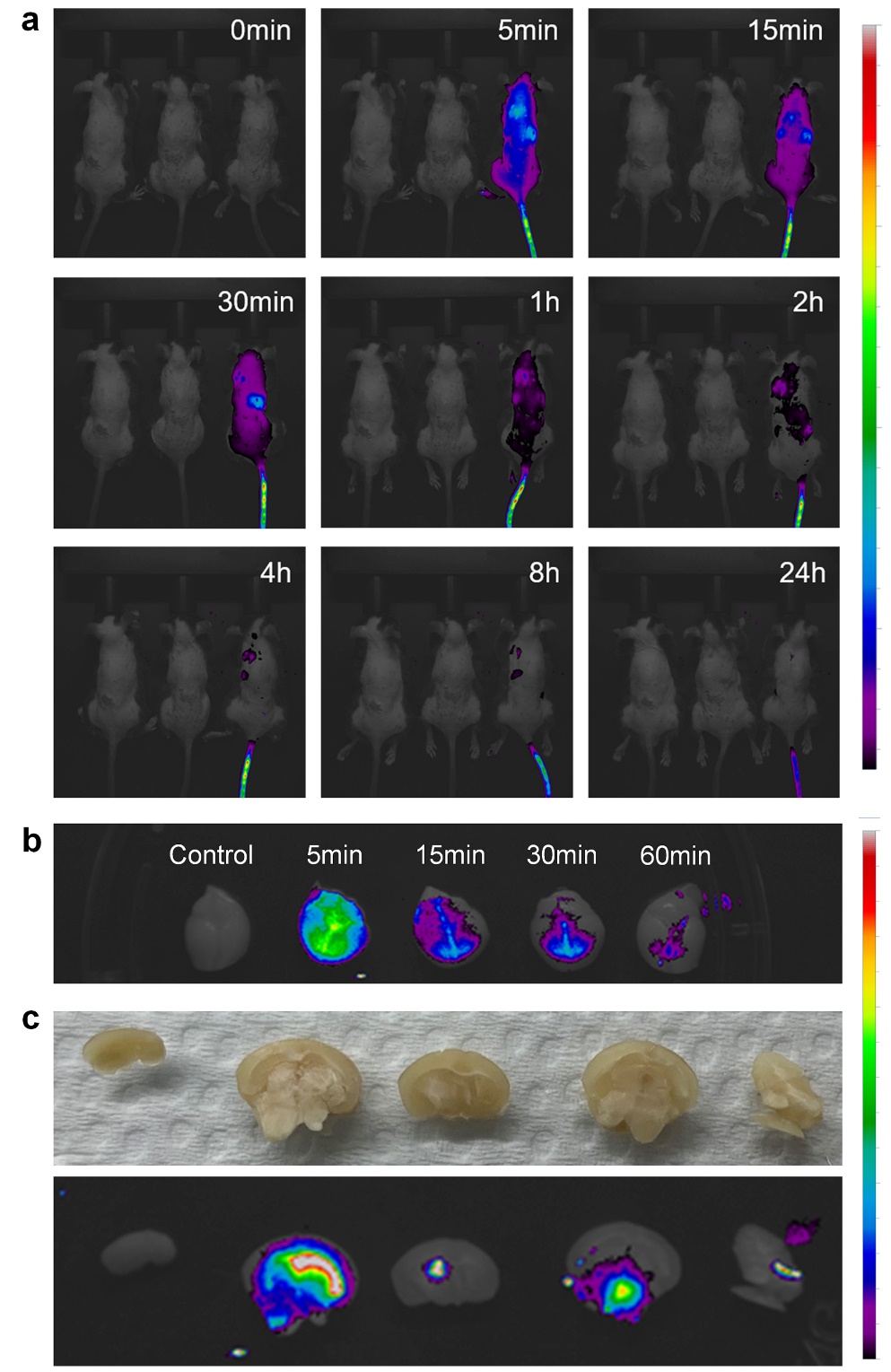


**Supplementary Figure 3. *In vivo* tracking of EVs post administration**.

(**a**) Mice were injected intravenously with EVs labeled with 1 mg/ml indocyanine green 1 day post MCAO. Shown are representative fluorescent images at different time points post administration. (**b**) Fluorescent images of mouse brains at different time points post intravenous administration of EVs. (**c**) Fluorescent images of serial coronal sections of mouse brains 5 min post intravenous administration of EVs. All fluorescent images were acquired using VISQUE Invivo Smart-LF small animal optical imaging system.


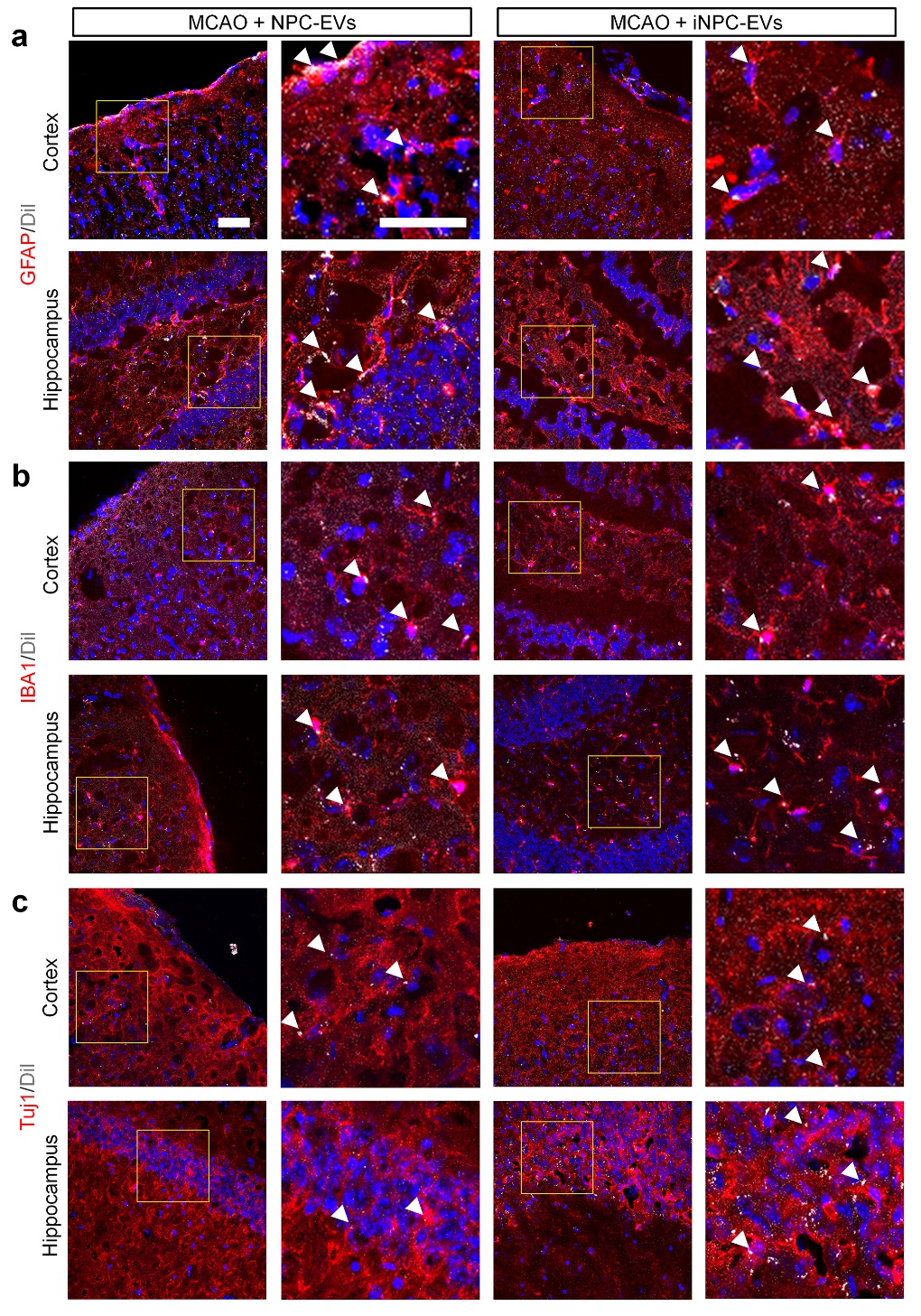


**Supplementary Figure 4. EVs are internalized by brain cells**.

Mice were injected intravenously with Dil-labeled EVs 1 day post MCAO. Brain tissues were collected 5 min after intravenous injection of EVs. (**a**) Representative confocal microscopy images of GFAP and Dil in the cortex and hippocampus. (**b**) Representative confocal microscopy images of Iba1 and Dil in the cortex and hippocampus. (**c**) Representative confocal microscopy images of Tuj1 and Dil in the cortex and hippocampus. Images at the right panels were high-magnification images of the corresponding small box area from the left panels in each group. Arrows indicate overlapping signals. Scale bar: 50 μm.


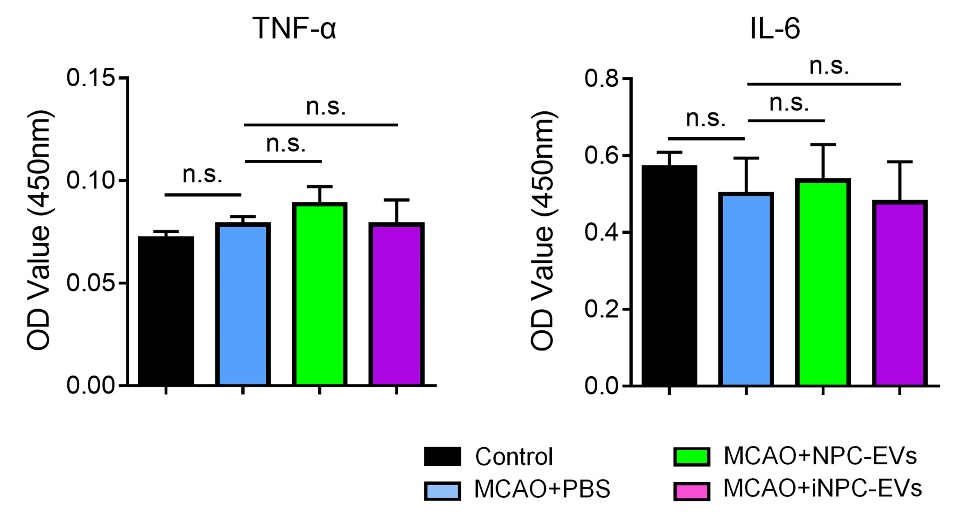


**Supplementary Figure 5. EVs have no effects on peripheral inflammation**.

The levels of pro-inflammatory cytokines TNF-α and IL-6 in mouse sera were determined by ELISA. Data were represented as mean ± s.d. from three independent experiments. ns denotes non-significance.


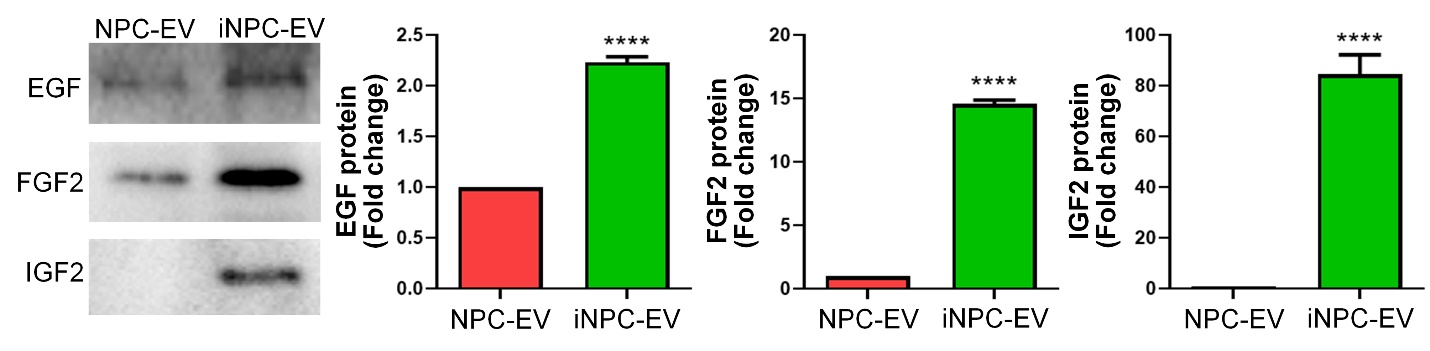


**Supplementary Figure 6. iNPC-EVs contain high levels of growth factors**.

The representative western blots showing the expression of EGF, FGF2, and IGF2 in 30 μg NPC- and iNPC-EVs (left). Densitometric quantifications were presented on the right. The EV lysate concentrations were determined by BCA Protein Assay Kit. Data were represented as mean ± s.d. from three independent experiments. **** denotes *p* < 0.0001.


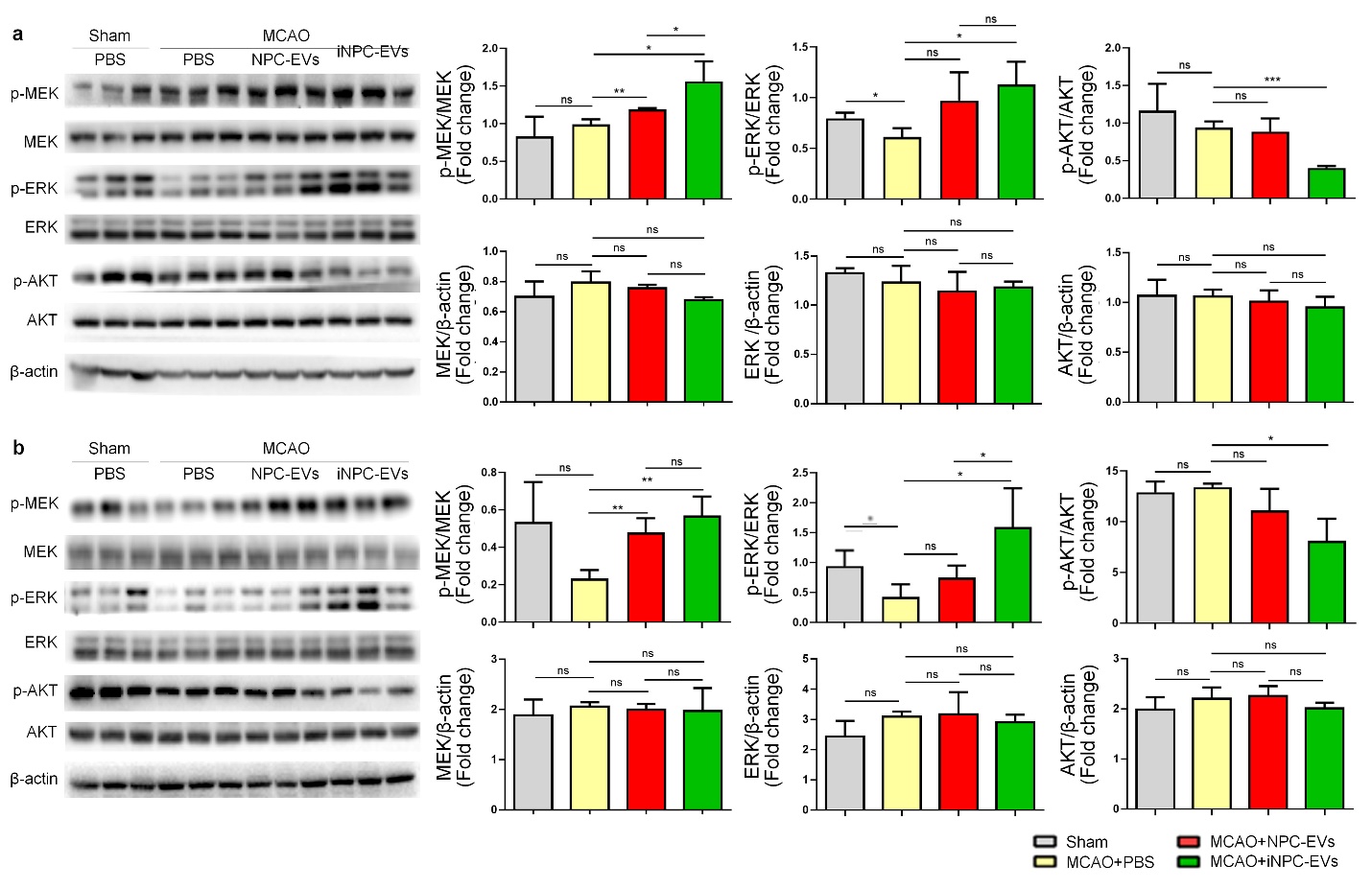


**Supplementary Figure 7. iNPC-EVs reverse the impaired ERK phosphorylation in MCAO mouse brains**.

(**a**) The representative western blots showing the expression of p-MEK, p-ERK, and p-AKT in hippocampal brain tissues of mice (left). Densitometric quantifications were presented on the right (n=3). (**b**) The representative western blots showing the expression of p-MEK, p-ERK, and p-AKT in cortical brain tissues of mice (left). Densitometric quantifications were presented on the right (n=3). Data were represented as mean ± s.d. from three independent experiments. * and ** denote *p* < 0.05 and *p* < 0.01, respectively. ns denotes non-significance.


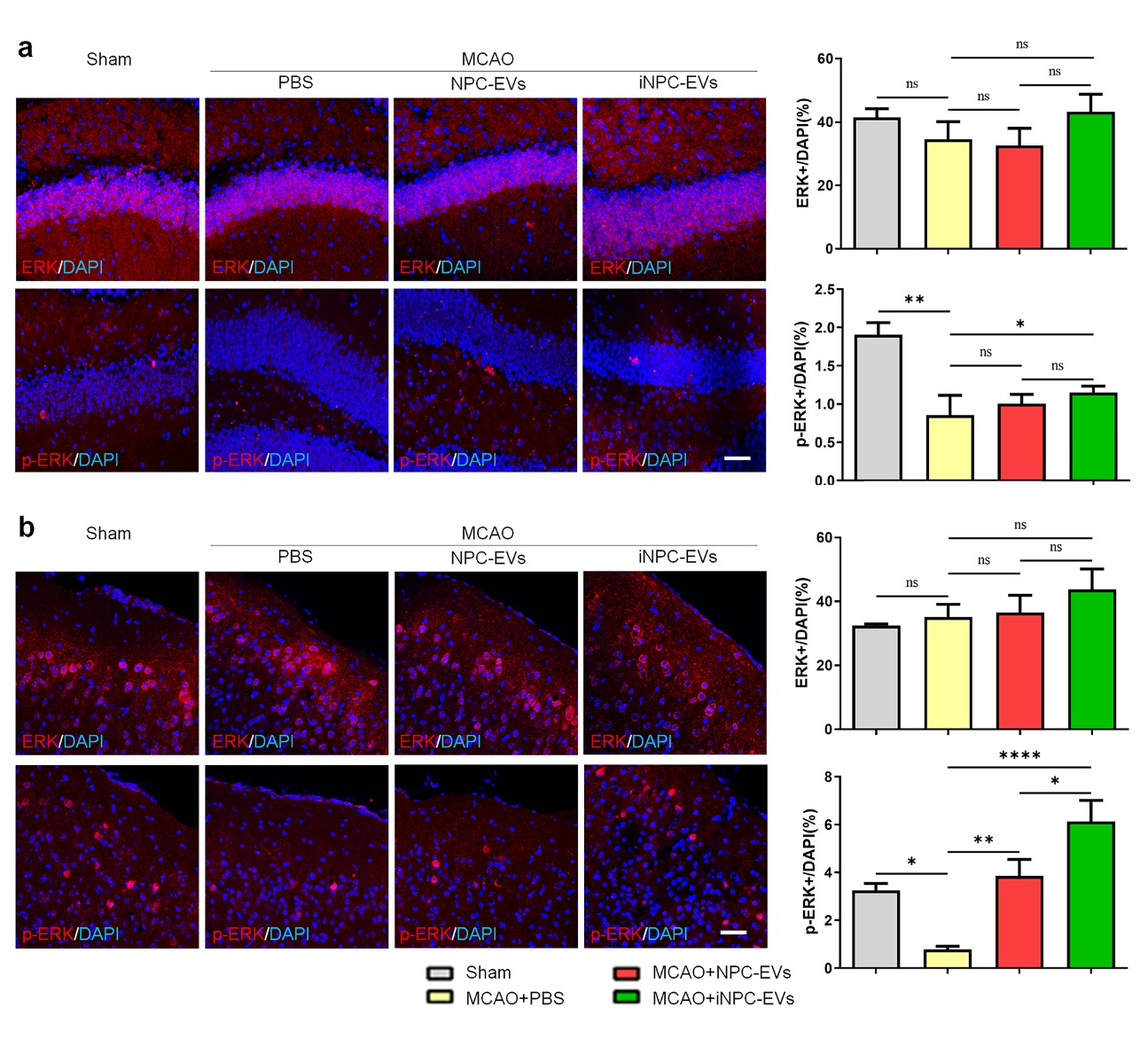


**Supplementary Figure 8. iNPC-EVs rescue ERK phosphorylation in MCAO mouse brains**.

(**a**) Representative confocal microscopy images of ERK and p-ERK in the hippocampus. Proportions of cells with ERK and p-ERK immunoreactivities in each group were given on the right panel (n=4). (**b**) Representative confocal microscopy images of ERK and p-ERK in peri-infarct cortex. Proportions of cells with ERK and p-ERK immunoreactivities in each group were given on the right panel (n=4). Data were represented as mean ± s.d. from three independent experiments. *, **, and **** denote *p* < 0.05, *p* < 0.01, and *p* < 0.0001, respectively. ns denotes non-significance. Scale bar: 50 μm.

| Gene | Sequence | Size (bp) | Accession N. |
| --- | --- | --- | --- |
| *Bcl2* | 5’-TACGAGTGGGATGCTGGAGA-3’  5’-CGGTAGCGACGAGAGAAGTC-3’ | 236 | NM_009741.5 |
| *Casp3* | 5’-GCAGAGCCATGGGCACATCT-3’  5’-GCCCTGGCCCTTTCGTTTCT-3’ | 294 | NM_009810.3 |
| *Casp8* | 5’-TTTCAGCAAGGCCCGGGAAG-3’  5’-GACACCCTTGTCACCGTGGG-3’ | 238 | NM_009812.2 |
| *GAPDH* | 5’-CATGTTCCAGTATGACTCCACTC-3’  5’-GGCCTCACCCCATTTGATGT-3’ | 136 | NM_001289726.1 |

**Supplementary Table 1. List of specific primers.**

**Materials and Methods**

**Mice**

C57BL/6 mice were housed and bred in the Comparative Medicine animal facilities of Tongji University School of Medicine. All procedures were conducted according to protocols approved by the Institutional Animal Care and Use Committee of Tongji University School of Medicine (reference number: SYXK (HU) 2014-0026).

**Isolation and enrichment of mouse NPCs**

NPCs were isolated from mouse fetal brain tissue as previously described [4]. Briefly, cortical tissues were isolated from embryonic day 13.5 mice, triturated 15-20 times, and filtered through 40 μm filter. Single cells were cultured in substrate-free tissue culture flasks with NPC culture medium containing NeuroCult® NSC Basal Medium (Stem Cell Technologies), NeuroCult® NSC Proliferation Supplements (Stem Cell Technologies), 20 ng/mL FGF2 (BioWalkersville), 20 ng/mL EGF (BioWalkersville) and 2 μg/mL heparin (Sigma), N2 supplement (Gibco), 2 mM L-glutamine (ThermoFisher), 100 U/ml penicillin & streptomycin (ThermoFisher) to generate neurospheres. Primary neurospheres were harvested, centrifuged at 200 g for 5 min to remove flowing cells, dissociated into single cells by Accutase (Sigma) treatment for 5 minat 37 °C, and re-plated for next round of neurosphere formation. NSCs were enriched after three rounds of neurosphere formation.

**Isolation of EVs**

Conditioned medium was collected 24 hours when enriched NPCs and iNPCs were cultured in NPC culture medium. EVs were then isolated from conditioned medium as previously described [5]. Briefly, conditioned media was first centrifuged at 300 g for 10 min to eliminate flowing cells, at 3,000 g for 20 min to eliminate cellular debris, at 10,000 g for 30 min to eliminate intracellular organelles, and then at 100,000 g for 2 h to precipitate EVs. All steps of centrifugation were performed at 4 °C. EVs were resuspended in PBS and stored in -80 °C until required.

**TTC assessment of infarct size**

TTC assessment was performed as previously described [1]. Mice were killed at 30 minutes and 60 min after MCAO. The brains were rapidly removed, frozen at -20°C for 30 min. 2 mm thick coronal sections of the brain were then cut in a rat brain matrix starting at the frontal pole. The sections were incubated with 2% triphenyltetrazolium chloride (TTC) in dark for 30 min at 37°C. After this, sections were fixed in 4% buffered formaldehyde solution for 20 min for imaging.

**Nanoparticle Tracking Analysis (NTA)**

The size and number of EVs were determined as previously described [2]. Briefly, 20 μl EVs suspension was diluted into 1 ml solution and then used for NanoSight analysis. NTA was done on NanoSight NS300 system (Malvern Instruments, UK) with a sCMOS camera. The conditions of the measurements were set at 25 °C, 1 cP viscosity, 25 sec per capture frame and 60 sec for measurement time. Three individual measurements were performed for measuring sizes and concentrations of EVs.

**Middle cerebral artery occlusion (MCAO)**

MCAO was achieved by transient middle cerebral artery occlusion (tMCAO) as previously described and adopted for mice [1]. Mice were anaesthetized with 4% chloralhydrate. Rectal temperature was monitored continuously and maintained at 37.0 ± 0.5°C with heating pads. The right common carotid artery (CCA) and internal carotid artery (ICA) were isolated and separated from the vagus nerve. 0.16 mm monofilament suture with a kink 10 ± 1 mm from its rounded polylysine-coated tip was introduced from CCA into ICA. After 30 min of occlusion, the suture was removed to allow reperfusion. Sham-operated mice underwent identical surgery except that the suture was not inserted.

**Adhesive Removal Test**

Small adhesive stimuli (20 mm^2^) were placed on the both sides of the mouse forelimbs. After being returned to their home cages, mice contacted and removed the stimuli one at a time using their teeth. The times to contact and remove the stimuli were recorded for each of 3 trials. Each animal (n = 8/group) was tested 7 and 14 days after MCAO. On each experimental day, the average performance of 3 trials was calculated and used for later statistical analysis.

**Hanging wire test**

Forelimb coordination was analyzed by hanging wire test. Briefly, mice were suspended by the forelimbs on a wire (45 cm long and 0.3 cm diameter) stretched between two posts 40 cm above a foam pillow. Each mouse was given three trails and the score was given based on the performance of each mouse. Score 0: fell off; score 1: hung onto the wire by two forepaws; score 2: hung onto the wire by two forepaws, but also attempted to climb onto the wire; score 3: hung onto the wire by two forepaws plus one or both hindpaws around the wire; score 4: hung onto the wire by all four paws plus tail wrapped; score 5: escaped. Inter-trial intervals for all animals were 30 minutes.

**Rotarod test**

Rotarod test was performed by placing the mice onto a rod. The rod was then rotated at 4 rpm at the beginning and the speed was increased by 1 rpm every 10 sec. The maximum speed is 40 rpm. The length of time for which mice stayed on the rod (to a maximum of 10 minutes) was recorded as an indicator of its grasping power. 3 trials were performed with 20 minute intervals, and the best result was recorded and used for later statistical anal.

**Morris Water Maze (MWM) test**

MWM was performed as previously described [3]. Briefly, mice were introduced into a circular, water-filled tank which was equally divided into four quadrants. Visual cues were placed around the pool in plain sight of the mouse to flag the submerged platform. Various parameters of mouse movement were recorded, including the time spent in each quadrant of the pool, the time taken to reach the platform (escape latency), and the total distance travelled. For each trial, the mouse was allowed no more than 60 sec to find the submerged platform before they were guided to the platform, removed from water, towel dried, and returned to their cage. Each mouse completed 4 trials per day during the 6-day training phase. One day after the training, the probe test was conducted. The platform was removed and each mouse was given 60 sec to swim in the water. The swimming was videotaped and analyzed by Ethovision XT (Noldus, Netherlands).

**Protein extraction and western blotting**

Brains tissues were removed from euthanized mice and homogenized by a homogenizer in the M-PER Protein Extraction Buffer (Pierce) containing a protease inhibitor cocktail (Sigma). Protein concentrations were determined with a BCA Protein Assay Kit (Pierce). Proteins (5-10 μg) from tissue lysates were separated by sodium dodecyl sulfate-polyacrylamide gel electrophoresis (SDS-PAGE) and electrophoretic transferred to polyvinyldifluoridene membranes (Millipore and Bio-Rad). Proteins were treated with purified primary antibodies for CD206 (mouse, cat#AF2535, R&D Systems, 1:500), Cox2 (rabbit, cat#ab15191, Abcam, 1:100) or β-actin (Sigma) overnight at 4°C followed by a horseradish peroxidase-linked secondary anti-rabbit or anti-mouse antibody (Cell Signaling Technologies, 1:10,000). Antigen-antibody complexes were visualized by Pierce ECL Western Blotting Substrate (Thermo Fisher Scientific, Waltham, MA). For data quantification, films were scanned with a CanonScan 9950F scanner and the acquired images were analyzed on a Macintosh computer using the free public domain NIH ImageJ program (at <http://rsb.info.nih.gov/nih-image/>).

**Immunocytochemistry**

Immunocytochemistry was done as previously described [1]. Briefly, tissue sections were fixed in 4% paraformaldehyde (Sigma) for 15 min at room temperature (RT). Post 3 times of PBS washes, sections were incubated with permeabilizing and blocking buffer containing 5% goat serum (Vector Laboratories) and 0.2% Triton X-100 (Bio-Rad) in PBS for 1 h at RT. Tissue sections were then incubated with primary antibody for Ki67 (rabbit, cat# 9129S, CST, 1:400), Sox2 (rabbit, cat# ab97959, Abcam, 1:200), DCX (rabbit, cat# 4604S, CST, 1:400), GFAP (Sigma, cat #AB5541, chicken, 1:400), Tuj1 (Sigma, cat# T2200, rabbit, 1:100), NeuN (mouse, cat# MAB377, Millipore, 1:200), or Iba1 (rabbit, cat# 234003, Synaptic system, 1:500) overnight at 4°C. The next day, tissue sections were incubated with secondary antibodies (Molecular Probes) for 1 h at RT after washing with PBS for 3 times. Sections were mounted using Vecta-Shield (Vector Laboratories). Images were taken using a Zeiss AX10 fluorescence microscope accompanied with ZEN 2.3 (blue edition) software. For quantification of immunoreactivity positive cell density, the hippocampus, peri-infarct cortex, and SVZ were imaged at ×20 magnification. Three coronal sections spanning the cortex and hippocampus at different depths on the rostro-caudal axis were analyzed for each animal. Four to five images were captured on matching areas of selected brain regions per section. The numbers of immunoreactivity positive cells were quantified by ImageJ and were divided by those of total cells in the area. Values from each section were averaged to acquire a mean immunoreactivity positive cell density for each animal.

**Quantitative reverse transcription-Polymerase Chain Reaction (RT-qPCR)**

The mRNA was isolated from brain tissues using RNeasy mini kit (Qiagen) according to the manufacturer’s instructions. Genomic DNA was removed and cDNA was synthesized using DNase I digestion kit (Qiagen) and miScript II reverse transcription kit (Qiagen), respectively. Transcripts were amplified using gene-specific primer (**Supplemental Table 1**) and SYBR green PCR kit (Qiagen) with the ABI7500 (Applied Biosystems). All RT-qPCR results measured each sample in triplicate and no-template blanks were used for negative controls. Amplification curves and gene expression were normalized to the house-keeping gene *Gapdh*.

**Terminal deoxynucleotidyltransferase dUTP nick end labeling (TUNEL) Assay**

TUNEL assay was done as previously described [4]. Briefly, after two times washing with PBS, the mouse brain sections were treated with a mixture of Label Solution and Enzyme Solution (In Situ Cell Death Detection Kit, TMR red, Sigma) for 1 hour at 37°C. Label Solution without Enzyme Solution was used as negative control. The DNase I recombinant (1000 U/ml) treated mouse brain sections (10 minutes incubation at RT) were used as positive control. The mouse brain sections were then washed twice in PBS. The mouse brain sections were mounted using VectaShield (Vector Labroatories) and processed to microscopy using a Zeiss AX10 fluorescence microscope accompanied with ZEN 2.3 (blue edition) software.

**Statistical analyses**

The statistical difference between two independent groups was analyzed with the unpaired Student’s *t*-test, and that among more than two groups was assessed with the parametric one-way ANOVA with post-hoc Bonferroni test. Data were shown as mean ± s.d., and significance was determined as *p* < 0.05.

**References**

1. Gao G., Li C., Zhu J., Wang Y., Huang Y., Zhao S., et al., Glutaminase 1 Regulates Neuroinflammation After Cerebral Ischemia Through Enhancing Microglial Activation and Pro-Inflammatory Exosome Release. *Front Immunol*, (2020) **11**: p. 161. DOI: 10.3389/fimmu.2020.00161.

2. Gao G., Zhao S., Xia X., Li C., Li C., Ji C., et al., Glutaminase C Regulates Microglial Activation and Pro-inflammatory Exosome Release: Relevance to the Pathogenesis of Alzheimer's Disease. *Front Cell Neurosci*, (2019) **13**: p. 264. DOI: 10.3389/fncel.2019.00264.

3. Wang Y., Li Y., Zhao R., Wu B., Lanoha B., Tong Z., et al., Glutaminase C overexpression in the brain induces learning deficits, synaptic dysfunctions, and neuroinflammation in mice. *Brain Behav Immun*, (2017) **66**: p. 135-145. DOI: 10.1016/j.bbi.2017.06.007.

4. Xia X., Lu H., Li C., Huang Y., Wang Y., Yang X., et al., miR-106b regulates the proliferation and differentiation of neural stem/progenitor cells through Tp53inp1-Tp53-Cdkn1a axis. *Stem Cell Res Ther*, (2019) **10**(1): p. 282. DOI: 10.1186/s13287-019-1387-6.

5. Levy E., Exosomes in the Diseased Brain: First Insights from In vivo Studies. *Front Neurosci*, (2017) **11**: p. 142. DOI: 10.3389/fnins.2017.00142.
